# Philadelphia Anatomical Rooms

**Published:** 1860-03

**Authors:** 


Philadelphia Anatomical Rooms.—The class of Dr. Agnew, the accomplished superintendent of these apartments, amounted this winter to two hundred and forty-six, the largest number of pupils, probably, ever assembled in the amphitheatre of any private teacher in the world.

